# *In vitro* activity of colistin in antimicrobial combination against carbapenem-resistant *Acinetobacter baumannii* isolated from patients with ventilator-associated pneumonia in Vietnam

**DOI:** 10.1099/jmm.0.000137

**Published:** 2015-10

**Authors:** Vien Le Minh, Nguyen Thi Khanh Nhu, Voong Vinh Phat, Corinne Thompson, Nguyen Phu Huong Lan, Tran Vu Thieu Nga, Pham Thi Thanh Tam, Ha Thanh Tuyen, Tran Do Hoang Nhu, Nguyen Van Hao, Huynh Thi Loan, Lam Minh Yen, Christopher M. Parry, Ho Dang Trung Nghia, James I. Campbell, Tran Tinh Hien, Louise Thwaites, Guy Thwaites, Nguyen Van Vinh Chau, Stephen Baker

**Affiliations:** ^1^​ Hospital for Tropical Diseases, Wellcome Trust Major Overseas Programme, Oxford University Clinical Research Unit, Ho Chi Minh City, Vietnam; ^2^​ Division of Infectious Diseases, Department of Medicine, University of California San Francisco, CA, USA; ^3^​ School of Chemistry and Molecular Biosciences, University of Queensland, Brisbane, Queensland, Australia; ^4^​ Centre for Tropical Medicine, Nuffield Department of Clinical Medicine, Oxford University, UK; ^5^​ Hospital for Tropical Diseases, Ho Chi Minh City, Vietnam; ^6^​ Clinical Sciences, Liverpool School of Tropical Medicine, Liverpool, UK; ^7^​ Pham Ngoc Thach University of Medicine, Ho Chi Minh City, Vietnam; ^8^​ London School of Hygiene and Tropical Medicine, London, UK

## Abstract

*Acinetobacter baumannii* has become one of the major infection threats in intensive care units (ICUs) globally. Since 2008, *A. baumannii* has been the leading cause of ventilator-associated pneumonia (VAP) in our ICU at an infectious disease hospital in southern Vietnam. The emergence of this pathogen in our setting is consistent with the persistence of a specific clone exhibiting resistance to carbapenems. Antimicrobial combinations may be a strategy to treat infections caused by these carbapenem-resistant *A. baumannii*. Therefore, we assessed potential antimicrobial combinations against local carbapenem-resistant *A. baumannii* by measuring *in vitro* interactions of colistin with four antimicrobials that are locally certified for treating VAP. We first performed antimicrobial susceptibility testing and multilocus variable number tandem repeat analysis (MLVA) genotyping on 74 *A. baumannii* isolated from quantitative tracheal aspirates from patients with VAP over an 18-month period. These 74 isolates could be subdivided into 21 main clusters by MLVA and >80 % were resistant to carbapenems. We selected 56 representative isolates for *in vitro* combination synergy testing. Synergy was observed in four (7 %), seven (13 %), 20 (36 %) and 38 (68 %) isolates with combinations of colistin with ceftazidime, ceftriaxone, imipenem and meropenem, respectively. Notably, more carbapenem-resistant *A. baumannii* isolates (36/43; 84 %) exhibited synergistic activity with a combination of colistin and meropenem than carbapenem-susceptible *A. baumannii* isolates (2/13; 15 %) (*P* = 0.023; Fisher's exact test). Our findings suggest that combinations of colistin and meropenem should be considered when treating carbapenem-resistant *A. baumannii* infections in Vietnam, and we advocate clinical trials investigating combination therapy for VAP.

## Introduction

*Acinetobacter baumannii* has emerged as one of the most important Gram-negative nosocomial pathogens affecting critically ill patients, and is one of the most common causes of ventilator-associated pneumonia (VAP) worldwide (Taylor *et al.*, 2012; Schultsz *et al.*, 2013). The treatment of *A. baumannii* infections is complicated because of increasing resistance to antimicrobial agents including carbapenems. Outbreaks of healthcare-associated infections caused by carbapenem-resistant *A. baumannii* have been reported, and carbapenem-resistant organisms are now widespread (Kim *et al.*, 2012; McGrath *et al.*, 2011; Song *et al.*, 2011). As a consequence of carbapenem resistance, the polymyxin drug colistin is becoming an alternative treatment for antimicrobial-resistant *A. baumannii* infections (De Pascale *et al.*, 2010; Lim *et al.*, 2011a, b; Kofteridis *et al.*, 2010; Lin *et al.*, 2010; Nakwan *et al.*, 2011). However, colistin is also an imperfect alternative, and colistin-resistant *A. baumannii* are becoming more frequently reported (Adams *et al.*, 2009; López-Rojas *et al.*, 2011; Moffatt *et al.*, 2010, 2011; Park *et al.*, 2009; Rodriguez *et al.*, 2009; Soon *et al.*, 2011).

In the intensive care unit (ICU) at the Hospital for Tropical Diseases (HTD), Ho Chi Minh City (HCMC), Vietnam, an apparent clonal replacement of a carbapenem-resistant *A. baumannii* strain in 2008 (Nhu *et al.*, 2014) has led to colistin being used as the first-line treatment for VAP caused by these infections. If these multidrug-resistant (MDR) *A. baumannii* isolates also become colistin resistant, they will be untreatable with locally available antimicrobials. Therefore, we hypothesize that antimicrobial combinations may be a therapeutic strategy that improves outcome against these MDR *A. baumannii* (Gordon *et al.*, 2010; Kiratisin *et al.*, 2010; Lim *et al.*, 2011b; Sheng *et al.*, 2011; Sopirala *et al.*, 2010; Tan *et al.*, 2011; Wareham *et al.*, 2011). We investigated the *in vitro* activity of colistin in combination with a variety of commonly used antimicrobial agents against carbapenem-resistant *A. baumannii* strains isolated from the ICU of the HTD. Our data showed that colistin used in combination with either third-generation cephalosporins or carbapenems may have a synergistic effect on treating infections caused by MDR *A. baumannii*.

## Methods

### Study site and patients

This study was performed on bacterial isolates collected as part of diagnostic tests performed as a standard of care at our hospital. The data were anonymized before analysis and individual patient consent was not required. The site of the study was the HTD in HCMC in the south of Vietnam. HTD is a 550-bed hospital that serves as a primary and secondary facility for the surrounding local population in HCMC and as a tertiary referral centre for infectious diseases for the 17 southern provinces of the country; it has a catchment population of approximately 40 million people. Nearly 70 % of HTD admissions are resident in HCMC, with the remainder resident in the surrounding provinces. Patients without infectious diseases, including those with surgical requirements, tuberculosis, cancer, primary haematological disorders or immunosuppression (other than human immunodeficiency virus infection) are generally referred to other healthcare settings in the city.

### Sample collection and microbiological culture

All strains were isolated from tracheal aspirate (TA) specimens taken from patients with suspected VAP in the ICU of the HTD in HCMC from January 2011 to June 2012. The criteria for analysis were: admission to the ICU, intubated for mechanical ventilation (due to respiratory failure), with a TA collected because of suspected VAP. VAP is defined as: pneumonia where the patient was on mechanical ventilation for >2 days when pneumonia was recorded, with day 1 being the first day of mechanical ventilation. If the patient was admitted to or transferred to the ICU on a ventilator, the day of admission was considered as day 1.

TAs were collected according to the local standard operating procedures of HTD. Patients were pre-oxygenated and a standard 500 mm, 14-gauge tracheal aspiration catheter (Argyle Sherwood Medical) was attached to a 20 ml syringe filled with 20 ml sterile saline. The distal end was lubricated with sterile gel, introduced via the tracheostomy or endotracheal tube, and advanced until significant resistance was encountered. The saline was instilled over 10–15 s, the tube was then withdrawn 10–20 mm, the saline was immediately re-aspirated and the catheter was then removed. In total, 5–10 ml fluid was recovered. No further aspiration was attempted during removal of the catheter to avoid contamination with tracheal secretions. Samples were transported to the microbiology laboratory, placed in the fridge at 4 °C and processed within 2 h of collection. The TA samples were examined by a Gram stain and the aspirate fluid was diluted 1 : 1 with Sputasol (Oxoid) and incubated at 37 °C, with periodic agitation, until liquefaction. The sample was then diluted (1 : 1, 10^− 1^ and 10^− 2^) using maximum recovery diluent (Oxoid), and 20 μl of 1 : 1 diluent was inoculated onto blood agar and chocolate agar base plates. Additionally, 20 μl of the 10^− 1^ and 10^− 2^ dilutions was plated onto MacConkey medium and blood agar base (all media were supplied by Oxoid Unipath). Inoculated media were incubated at 37 °C and examined after 24 and 48 h of incubation. The threshold used to discriminate between infection and colonization was ≥ 1 × 10^5^ c.f.u. ml^− 1^ (i.e. >20 colonies on either medium from the 10^− 2^ dilution). Colonies above this threshold were identified using an in-house bacteriological identification (biochemical short-set) kit and/or by API 20E and API 20NE kits following the manufacturer's guidelines (bioMérieux).

*Acinetobacter* spp. identified using API 20NE were confirmed by a previously described PCR method to detect *bla*_OXA-51_ (Woodford *et al.*, 2006). PCR amplifications were performed in 20 μl reaction volumes, containing 1 μl template genomic DNA, 0.2 μM each primer, 2 U *Taq* DNA polymerase, 200 μM each dNTP and 1.5 mM MgCl_2_ in 1 ×  PCR buffer (all PCR reagents were supplied by Bioline). PCR amplifications were visualized on 1 % agarose gels under UV light after staining with ethidium bromide and were compared with the predicted sizes. All *A. baumannii* isolates were stored in glycerol at − 70 °C until the synergistic testing was performed.

### Antimicrobial susceptibility testing

Antimicrobial susceptibilities were determined at the time of isolation by the modified Bauer–Kirby disc diffusion method, as recommended by the CLSI guidelines (CLSI, 2012). The antimicrobials tested were piperacillin/tazobactam (100/10 μg), imipenem (10 μg), meropenem (10 μg), amikacin (30 μg), ceftriaxone (30 μg), ceftazidime (30 μg), ticarcillin/clavulanic acid (75/10 μg), cefepime (30 μg), levofloxacin (5 μg) and co-trimoxazole (1.25/23.75 μg). Mueller–Hinton agar and antimicrobial discs were purchased from Oxoid. For colistin, meropenem, imipenem, ceftazidime and ceftriaxone, MICs were determined by E-test based on the manufacturer's recommendations (bioMérieux). The results were interpreted as resistant or sensitive according to current CLSI guidelines (CLSI, 2012). *Escherichia coli* ATCC 25922 was used as the control for these assays. For isolates that had an MIC above the range of the E-test, MICs were determined by the broth dilution method (Wiegand *et al.*, 2008). Briefly, 5 × 10^5^ c.f.u. (ml bacteria)^− 1^ were inoculated into a series of Mueller–Hinton broths containing two-fold dilutions of the antimicrobial agent. Following inoculation, the broth was incubated at 37 °C for 18–24 h. The MIC was defined as the lowest concentration of antimicrobial that inhibited the growth of bacteria.

### Multilocus variable number tandem repeat (MLVA) genotyping

DNA was extracted from the 74 *A. baumannii* selected for further analysis using a Wizard Genomic DNA Extraction kit (Promega). The quality and concentration of the DNA were assessed using a Nanodrop Bioanalyser spectrophotometer (Thermo Scientific). Genomic DNA from all strains was standardized to a concentration of 25 ng μl^− 1^ for further use. The 74 selected strains of *A. baumannii* were genotyped using the MLVA method developed by Pourcel *et al.* (2011) with some modifications. Briefly, genomic DNA from each of the 74 *A. baumannii* was subjected to three multiplex PCR amplifications (in a total volume of 50 μl), in which the annealing temperature was set at 50 °C. The sizes of the amplicons [corresponding to eight variable number tandem repeats (VNTRs)] at each locus were determined by capillary electrophoresis fragment analysis using an ABI 3130 XL capillary electrophoresis system (Applied Biosystems). For fragment analysis, 0.5 μl PCR amplicon was mixed with 9.25 μl Hi-Di Formamide and 0.25 μl GeneScan LIZ500 size standard (Applied Biosystems). The mixture was incubated for 3 min at 95 °C, chilled for 10 min and analysed. Resulting fragment analysis data were analysed using GeneMapper v.4.0 (Applied Biosystems). Furthermore, to determine the number of repeating units, the different-sized amplicons at each locus were DNA sequenced. PCR amplicons were purified using a PCR purification kit (Qiagen) and sequenced using a BigDye Terminator Sequencing kit (Applied Biosystems). All data were analysed using a numeric coefficient in BioNumerics software (Applied Maths) and trees were drawn using Dendroscope v.2.3.

### Synergy testing by chequerboard assay

The *in vitro* activity of colistin in combination with meropenem, imipenem, ceftazidime or ceftriaxone was assessed in a microtitre plate chequerboard assay (Petersen *et al.*, 2006). All antimicrobials were obtained from Sigma-Aldrich and stock solutions of 10 000 mg l^− 1^ were prepared in sterile water and stored at − 20 °C until use. The range of final working concentrations of each antimicrobial varied and depended on the MIC of each strain. The concentration range for colistin was 0.03–2 mg l^− 1^, for carbapenem was 0.13–8 μg ml^− 1^ and the for the cephalosporins was 1–64 mg l^− 1^. The final concentration of the test strain was approximately 5 × 10^5^ c.f.u. ml^− 1^ in a final volume of 100 μl in each well. The *A. baumannii* were incubated at 37 °C for 18–24 h. The chequerboard titration of each combination was carefully performed with positive (with bacteria) and negative (without bacteria) controls.

The fractional inhibitory concentration (FIC) to determine synergy between antimicrobial agents was calculated as follows: FIC of drug A = (MIC of drug A in combination)/(MIC of drug A alone); FIC of drug B = (MIC of drug B in combination)/(MIC of drug B alone). The FIC index (FICI) was defined as the FIC of drug A added to the FIC of drug B. The FICI was interpreted as follows: synergistic, FICI ≤ 0.5; indifferent, 0.5 < FICI < 4; and antagonistic, FICI ≥ 4.0 (Hall *et al.*, 1983). The assay was repeated on three occasions for each isolate.

## Results

### Microbial culture of TAs

From January 2011 to June 2012, there were 156 patients with suspected VAP in the ICU at HTD in HCMC. From these, a total of 228 TAs were collected for bacteriological assessment, of which 166 (72.8 %) produced a significant bacterial culture result (defined as ≥ 10 ^5^ c.f.u. ml^− 1^ of a potential VAP pathogen). The predominant bacterial species were the *Acinetobacter* spp., accounting for nearly half of the isolated bacteria (94/195), of which 74 (78.7 %) were identified as *A. baumannii* by PCR amplification of the *bla*_OXA-51_ gene.

The 74 *A. baumannii* strains were subjected to antimicrobial susceptibility testing against 11 antimicrobials (Table 1). The *A. baumannii* isolates exhibited substantial antimicrobial resistance, with 54 % of the isolates resistant to all antimicrobials tested except colistin. According to current CLSI breakpoints, colistin was the only active agent against all *A. baumannii* isolates, while >80 % of the *A. baumannii* isolates were resistant to at least three different antimicrobial classes, including carbapenems, cephalosporins and fluoroquinolones. Notably, the MIC_90_ for meropenem and imipenem was >32 mg l^− 1^, and was >256 mg l^− 1^ for ceftriaxone and ceftazidime.

**Table 1.** Antimicrobial susceptibilities of 74 *A. baumannii* isolates from VAP patients CO, colistin; IMP, imipenem; MEP, meropenem; CAZ, ceftazidime; CRO, ceftriaxone; TZP, piperacillin/tazobactam; AK, amikacin; TCC, ticarcillin/clavulanic acid; SXT, co-trimoxazole; FEP, cefepime; LEV, levofloxacin; nt, not tested.

Antimicrobial	Resistant (%)^*^	MIC (mg l^− 1^)		
		Range	MIC_50_	MIC_90_
CO	0 (0 %)	0.047–0.75	0.19	0.38
IMP	61 (82 %)	0.25–512	64	64
MEP	62 (84 %)	0.19–128	32	64
CAZ	64 (86 %)	2–4096	256	512
CRO	65 (88 %)	2–2048	1024	2048
FEP	69 (93 %)	nt	nt	nt
TZP	66 (89 %)	nt	nt	nt
AK	61 (82 %)	nt	nt	nt
TCC	66 (89 %)	nt	nt	nt
SXT	60 (80 %)	nt	nt	nt
FEP	69 (93 %)	nt	nt	nt
LEV	60 (80 %)	nt	nt	nt

* Resistance breakpoints (mg l^− 1^): colistin, 4; imipenem, 16; meropenem, 16; ceftazidime, 32; ceftriaxone, 64.

### Genotyping of *A. baumannii*

**Fig. 1.**
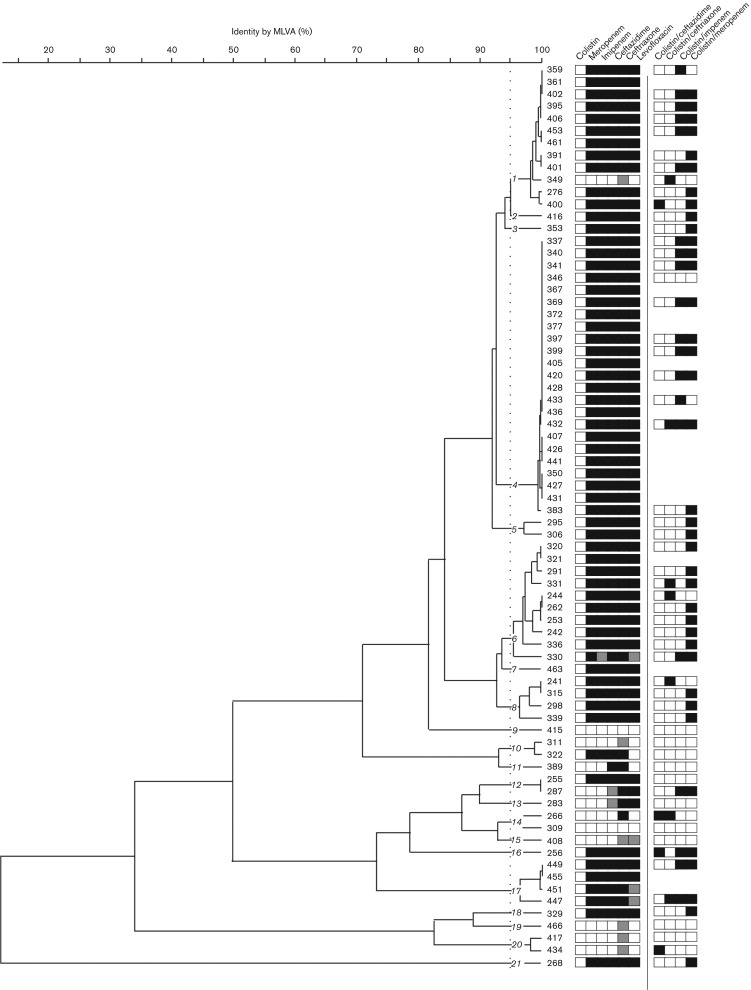
MLVA genotyping of 74 Vietnamese *A. baumannii* isolates. Dendrogram created from MLVA using eight VNTR loci from 74 *A. baumannii* strains isolated from the ICU at the HTD in HCMC between January 2011 and June 2012.The strain numbers are shown to the right of the dendrogram and the 21 strain clusters are highlighted (arbitrary cut-off of >95 % identity). Associated metadata include susceptibility to colistin, meropenem, imipenem, ceftazidime, ceftriaxone and levofloxacin (white, susceptible; grey, intermediate; black, resistant) and synergy between colistin and ceftazidime, ceftriaxone, imipenem or meropenem (black, synergism; white, indifference).

MLVA was performed on eight VNTR loci to determine relatedness among the 74 *A. baumannii* isolates associated with VAP. With an arbitrary cut-off set at 95 % similarity, the MLVA patterns classified these isolates into 21 discrete clusters (Fig. 1). Three main groups were observed (labelled 1, 4 and 6 in Fig. 1). However, one clone (group 4) dominated and was responsible for a third of all the VAP cases caused by *A. baumannii* during this period. All of these isolates were resistant to meropenem, imipenem, ceftazidime, ceftriaxone and levofloxacin. A total of 56 *A. baumannii* isolates were selected for synergy testing with an array of antimicrobials through comparison of antimicrobial and MVLA profiles in order to cover a range of genotypes. The selected strains and their associated resistance profile are summarized in Fig. 1.

### *In vitro* synergy testing of colistin in combination with other antimicrobials

The 56 *A. baumannii* isolates were subjected to *in vitro* synergy testing using a chequerboard method to identify potential activity between colistin and the other antimicrobial agents currently available and licensed for treating VAP in Vietnam: meropenem, ceftazidime, ceftriaxone and imipenem. Data from the 224 combination assays are shown in Table 2 and Fig. 1. Sixty-nine (31 %) of the assays demonstrated a synergistic interaction, with the remaining 155 (69 %) assays showing indifferent interactions. No antagonism was identified with any combinations of antimicrobials (Table 2). Synergistic activity was observed between colistin and ceftazidime (4/56 isolates; 7 %), colistin and ceftriaxone (7/56 isolates; 13 %), and colistin and imipenem (20/56 isolates; 36 %). The greatest degree of synergistic activity was observed between colistin and meropenem, with 38/56 (68 %) demonstrating a decreased FICI.

**Table 2.** Antimicrobial combinations against *A. baumannii* using the chequerboard titration assay

Antimicrobial combination	No. tested	Nature of interaction [*n* (%)]^*^	
		Synergism	Indifference
Ceftazidime/colistin	56	4 (7)	52 (93)
Ceftriaxone/colistin	56	7 (13)	49 (87)
Imipenem/colistin	56	20 (36)	36 (64)
Meropenem/colistin	56	38 (68)	18 (32)
Total	224	69 (31)	155 (69)

* Interaction: FICI ≤ 0.5, synergism; 0.5 < FICI < 4, indifference.

To assess the potential impact of the combination of colistin and meropenem in treating carbapenem-resistant *A. baumannii* VAP infections, the data resulting from the synergy assays were stratified into two groups, carbapenem-resistant and carbapenem-susceptible isolates (Fig. 1, Table 3). Synergism was observed more frequently (in any colistin/antimicrobial combination) in the carbapenem-resistant *A. baumannii* isolates than in the carbapenem-susceptible isolates. Furthermore, there was a significant synergistic effect between colistin and meropenem in carbapenem-resistant *A. baumannii* (36/43; 84 %) compared with the carbapenem-susceptible isolates (2/13; 15 %) (*P* = 0.023, Fisher's exact test). Notably, synergy between colistin and carbapenems was observed in all the dominant MLVA groups (including group 4) and across a broad range of the MLVA subgroups.

**Table 3.** Antimicrobial combinations against carbapenem-resistant and -susceptible *A. baumannii*

Antimicrobial combination	Nature of interaction [*n* (%)] in carbapenem-resistant *A. baumannii* (*n* = 43)^*^		Nature of interaction [*n* (%)] in carbapenem-sensitive *A. baumannii* (*n* = 13)^*^	
	Synergism	Indifference	Synergism	Indifference
Ceftazidime/colistin	2 (5)	41 (95)	2 (15)	11 (85)
Ceftriaxone/colistin	5 (12)	38 (88)	2 (15)	11 (85)
Meropenem/colistin	36 (84)	7 (16)	2 (15)	11 (85)
Total	43 (33)	86 (67)	6 (15)	33 (85)

* Interaction: synergism, FICI ≤ 0.5; indifference, 0.5 < FICI < 4.

## Discussion

*A. baumannii* is a ubiquitous environmental organism that can cause a wide range of opportunistic infections in healthcare settings, including septicaemia, pneumonia, endocarditis, meningitis, skin infections, wound infections and urinary tract infections (Bergogne-Bérézin & Towner, 1996). Indeed *A. baumannii* is now widely acknowledged as an important cause of VAP (Choi *et al.*, 2010; Peleg *et al.*, 2008). *A. baumannii* is a substantial threat in critically ill patients in ICUs and other high-dependency healthcare units (Barnaud *et al.*, 2010; Jung *et al.*, 2010; McGrath *et al.*, 2011). In Vietnam, *A. baumannii* is recognized as a substantial problem in healthcare facilities, causing infections in intubated adults and critically ill children and neonates (Kruse *et al.*, 2013; Schultsz *et al.*, 2013; Tada *et al.*, 2013). We have found that the proportion of patients at our hospital infected with MDR *A. baumannii* has been steadily increasing over the last decade (Nhu *et al.*, 2014). The increase in *A. baumannii* in Vietnam has led to widespread use of polymyxins, particularly colistin, for the treatment of severe infections with this organism. Additional data from Vietnam is currently limited, but we speculate that this trend is widespread in other less-well-equipped healthcare settings across the country.

In this study, we determined the potential *in vitro* efficacy of colistin in combination with other antimicrobial agents against a range of *A. baumannii* isolated from VAP patients in our ICU. Several studies have evaluated the ability of other antimicrobial agents to induce synergy when combined with colistin *in vitro* (Kruse *et al.*, 2013; Paul *et al.*, 2004; Petrosillo *et al.*, 2008; Tada *et al.*, 2013; Zusman *et al.*, 2013). We assessed the synergistic potential of colistin with ceftazidime, ceftriaxone, imipenem and meropenem against 56 clinical isolates of *A. baumannii*, collected over an 18-month period. Our data showed that synergistic activity between colistin in combination with either cephalosporins (ceftazidime and ceftriaxone) or carbapenems (imipenem and meropenem) was highly variable. Overall, there was limited synergistic interaction between colistin and ceftazidime/ceftriaxone, with marked *in vitro* synergy between colistin and ceftazidime in only 7 % and ceftriaxone in 13 % of isolates. Synergy between colistin and third-generation cephalosporins has been reported previously for both MDR *A. baumannii* and *Pseudomonas aeruginosa* (Gunderson *et al.*, 2003; Kroeger *et al.*, 2007). In contrast, we found that synergy between colistin and carbapenems was more common, with 68 % of *A. baumannii* isolates exhibiting a synergistic effect between colistin and meropenem, and 36 % of isolates showing synergy between colistin and imipenem. The synergistic activity of colistin and meropenem was significantly more common in carbapenem-resistant *A. baumannii* isolates compared with carbapenem-susceptible *A. baumannii* isolates, with 84 versus 15 % of isolates displaying synergy, respectively. This difference in synergistic interaction was not observed with the combinations of colistin and ceftazidime, or colistin and ceftriaxone.

Our results support the findings of a recent systemic review and meta-analysis of *in vitro* synergy of polymyxins and carbapenems, which found significant advantages of combining meropenem or doripenem with colistin, rather than imipenem, for *A. baumannii* infections (Zusman *et al.*, 2013). Both meropenem and imipenem induce bacterial lysis in susceptible organisms by high-affinity binding to high-molecular-mass penicillin-binding proteins (Zhanel *et al.*, 1998). Meropenem has a higher affinity for penicillin-binding proteins than imipenem in Gram-negative organisms, which may account for the enhanced performance of meropenem in our chequerboard assays (Zhanel *et al.*, 1998). Further evidence for the potential value of colistin combination therapy arose from a clinical study that measured resistance development in colistin monotherapy compared with colistin combination therapy (Paul *et al.*, 2004). This study found that colistin resistance could develop in the first 24 h of treatment with colistin monotherapy, and that colistin resistance was suppressed and delayed by combination therapy.

The current study indicates that combinations of colistin with ceftazidime, ceftriaxone, imipenem or meropenem have *in vitro* synergistic activity against local carbapenem-resistant *A. baumannii* strains in Vietnam. We found that colistin/meropenem had the greatest potential synergistic effect. Antimicrobial combinations may improve outcome by broadening the spectrum of antimicrobial activity, minimizing the potential emergence of antimicrobial-resistant organisms and by achieving a stronger antimicrobial effect through synergy. We cannot assume *in vivo* efficacy due to potential variability in pharmacokinetic effects of these drugs in the host, and different bacterial and drug concentrations in the specific sites of infection. In addition, these *in vitro* studies did not examine bactericidal activity, which is particularly relevant with respect to colistin combination therapy. Clinical investigations are required to elucidate the mechanism responsible for this effect and to explore its therapeutic potential, and we suggest that a suitable combination of these drugs should be tested rigorously in clinical trials of infections caused by MDR *A. baumannii*.

## References

[jmm000137-Adams] Adams M. D. Nickel G. C. Bajaksouzian S. Lavender H. Murthy A. R. Jacobs M. R. Bonomo R. A. (2009). Resistance to colistin in *Acinetobacter baumannii* associated with mutations in the PmrAB two-component system Antimicrob Agents Chemother 53 3628–3634 10.1128/AAC.00284-09 . 19528270 PMC2737849

[jmm000137-Barnaud1] Barnaud G. Zihoune N. Ricard J. D. Hippeaux M. C. Eveillard M. Dreyfuss D. Branger C. (2010). Two sequential outbreaks caused by multidrug-resistant *Acinetobacter baumannii* isolates producing OXA-58 or OXA-72 oxacillinase in an intensive care unit in France J Hosp Infect 76 358–360 10.1016/j.jhin.2010.05.026 . 20692729

[jmm000137-Bergogne-Berezin1] Bergogne-Bérézin E. Towner K. J. (1996). *Acinetobacter* spp. as nosocomial pathogens: microbiological, clinical, and epidemiological features Clin Microbiol Rev 9 148–165 . 8964033 10.1128/cmr.9.2.148 PMC172888

[jmm000137-Choi1] Choi W. S. Kim S. H. Jeon E. G. Son M. H. Yoon Y. K. Kim J. Y. Kim M. J. Sohn J. W. Kim M. J. Park D. W. (2010). Nosocomial outbreak of carbapenem-resistant *Acinetobacter baumannii* in intensive care units and successful outbreak control program J Korean Med Sci 25 999–1004 10.3346/jkms.2010.25.7.999 . 20592889 PMC2890899

[jmm000137-CLSI1] CLSI (2012). Performance Standards for Antimicrobial Susceptibility Testing 20th informational supplement Wayne, PA Clinical and Laboratory Standards Institute.

[jmm000137-De1] De Pascale G. Pompucci A. Maviglia R. Spanu T. Bello G. Mangiola A. Scoppettuolo G. (2010). Successful treatment of multidrug-resistant *Acinetobacter baumannii* ventriculitis with intrathecal and intravenous colistin Minerva Anestesiol 76 957–960 . 20445494

[jmm000137-Gordon1] Gordon N. C. Png K. Wareham D. W. (2010). Potent synergy and sustained bactericidal activity of a vancomycin-colistin combination versus multidrug-resistant strains of *Acinetobacter baumannii* Antimicrob Agents Chemother 54 5316–5322 10.1128/AAC.00922-10 . 20876375 PMC2981237

[jmm000137-Gunderson1] Gunderson B. W. Ibrahim K. H. Hovde L. B. Fromm T. L. Reed M. D. Rotschafer J. C. (2003). Synergistic activity of colistin and ceftazidime against multiantibiotic-resistant *Pseudomonas aeruginosa* in an in vitro pharmacodynamic model Antimicrob Agents Chemother 47 905–909 10.1128/AAC.47.3.905-909.2003 . 12604520 PMC149291

[jmm000137-Hall1] Hall M. J. Middleton R. F. Westmacott D. (1983). The fractional inhibitory concentration (FIC) index as a measure of synergy J Antimicrob Chemother 11 427–433 10.1093/jac/11.5.427 . 6874629

[jmm000137-Jung1] Jung J. Y. Park M. S. Kim S. E. Park B. H. Son J. Y. Kim E. Y. Lim J. E. Lee S. K. Lee S. H. other authors (2010). Risk factors for multi-drug resistant *Acinetobacter baumannii* bacteremia in patients with colonization in the intensive care unit BMC Infect Dis 10 228 10.1186/1471-2334-10-228 . 20670453 PMC2921386

[jmm000137-Kim1] Kim Y. J. Kim S. I. Kim Y. R. Hong K. W. Wie S. H. Park Y. J. Jeong H. Kang M. W. (2012). Carbapenem-resistant *Acinetobacter baumannii*: diversity of resistant mechanisms and risk factors for infection Epidemiol Infect 140 137–145 10.1017/S0950268811000744 . 21554783

[jmm000137-Kiratisin1] Kiratisin P. Apisarnthanarak A. Kaewdaeng S. (2010). Synergistic activities between carbapenems and other antimicrobial agents against *Acinetobacter baumannii* including multidrug-resistant and extensively drug-resistant isolates Int J Antimicrob Agents 36 243–246 10.1016/j.ijantimicag.2010.04.011 . 20541913

[jmm000137-Kofteridis1] Kofteridis D. P. Alexopoulou C. Valachis A. Maraki S. Dimopoulou D. Georgopoulos D. Samonis G. (2010). Aerosolized plus intravenous colistin versus intravenous colistin alone for the treatment of ventilator-associated pneumonia: a matched case–control study Clin Infect Dis 51 1238–1244 10.1086/657242 . 20973727

[jmm000137-Kroeger1] Kroeger L. A. Hovde L. B. Mitropoulos I. F. Schafer J. Rotschafer J. C. (2007). Colistin methanesulfonate against multidrug-resistant *Acinetobacter baumannii* in an in vitro pharmacodynamic model Antimicrob Agents Chemother 51 3431–3433 10.1128/AAC.01433-06 . 17576842 PMC2043208

[jmm000137-Kruse1] Kruse A. Y. Thieu Chuong H. Phuong C. N. Duc T. Graff Stensballe L. Prag J. Kurtzhals J. Greisen G. Pedersen F. K. (2013). Neonatal bloodstream infections in a pediatric hospital in Vietnam: a cohort study J Trop Pediatr 59 483–488 10.1093/tropej/fmt056 . 23868576

[jmm000137-Lim1] Lim S. K. Lee S. O. Choi S. H. Choi J. P. Kim S. H. Jeong J. Y. Choi S. H. Woo J. H. Kim Y. S. (2011a). The outcomes of using colistin for treating multidrug resistant *Acinetobacter* species bloodstream infections J Korean Med Sci 26 325–331 10.3346/jkms.2011.26.3.325 . 21394298 PMC3051077

[jmm000137-Lim12] Lim T. P. Tan T. Y. Lee W. Sasikala S. Tan T. T. Hsu L. Y. Kwa A. L. (2011b). In-vitro activity of polymyxin B, rifampicin, tigecycline alone and in combination against carbapenem-resistant *Acinetobacter baumannii* in Singapore PLoS One 6 e18485 10.1371/journal.pone.0018485 . 21533030 PMC3080872

[jmm000137-Lin1] Lin C. C. Liu T. C. Kuo C. F. Liu C. P. Lee C. M. (2010). Aerosolized colistin for the treatment of multidrug-resistant *Acinetobacter baumannii* pneumonia: experience in a tertiary care hospital in northern Taiwan J Microbiol Immunol Infect 43 323–331 10.1016/S1684-1182(10)60050-3 . 20688293

[jmm000137-Lopez-Rojas1] López-Rojas R. Domínguez-Herrera J. McConnell M. J. Docobo-Peréz F. Smani Y. Fernández-Reyes M. Rivas L. Pachón J. (2011). Impaired virulence and in vivo fitness of colistin-resistant *Acinetobacter baumannii* J Infect Dis 203 545–548 10.1093/infdis/jiq086 . 21216865 PMC3071218

[jmm000137-McGrath1] McGrath E. J. Chopra T. Abdel-Haq N. Preney K. Koo W. Asmar B. I. Kaye K. S. (2011). An outbreak of carbapenem-resistant *Acinetobacter baumannii* infection in a neonatal intensive care unit: investigation and control Infect Control Hosp Epidemiol 32 34–41 10.1086/657669 . 21091204

[jmm000137-Moffatt1] Moffatt J. H. Harper M. Harrison P. Hale J. D. Vinogradov E. Seemann T. Henry R. Crane B. St Michael F. other authors (2010). Colistin resistance in *Acinetobacter baumannii* is mediated by complete loss of lipopolysaccharide production Antimicrob Agents Chemother 54 4971–4977 10.1128/AAC.00834-10 . 20855724 PMC2981238

[jmm000137-Moffatt12] Moffatt J. H. Harper M. Adler B. Nation R. L. Li J. Boyce J. D. (2011). Insertion sequence IS*Aba11* is involved in colistin resistance and loss of lipopolysaccharide in *Acinetobacter baumannii* Antimicrob Agents Chemother 55 3022–3024 10.1128/AAC.01732-10 . 21402838 PMC3101452

[jmm000137-Nakwan1] Nakwan N. Wannaro J. Thongmak T. Pornladnum P. Saksawad R. Nakwan N. Chokephaibulkit K. (2011). Safety in treatment of ventilator-associated pneumonia due to extensive drug-resistant *Acinetobacter baumannii* with aerosolized colistin in neonates: a preliminary report Pediatr Pulmonol 46 60–66 10.1002/ppul.21324 . 20812247

[jmm000137-Nhu1] Nhu N. T. Lan N. P. Campbell J. I. Parry C. M. Thompson C. Tuyen H. T. Hoang N. V. Tam P. T. Le V. M. other authors (2014). Emergence of carbapenem-resistant *Acinetobacter baumannii* as the major cause of ventilator-associated pneumonia in intensive care unit patients at an infectious disease hospital in southern Vietnam J Med Microbiol 63 1386–1394 10.1099/jmm.0.076646-0 . 25038137 PMC4170484

[jmm000137-Park1] Park Y. K. Jung S. I. Park K. H. Cheong H. S. Peck K. R. Song J. H. Ko K. S. (2009). Independent emergence of colistin-resistant *Acinetobacter* spp. isolates from Korea Diagn Microbiol Infect Dis 64 43–51 10.1016/j.diagmicrobio.2009.01.012 . 19362258

[jmm000137-Paul1] Paul M. Benuri-Silbiger I. Soares-Weiser K. Leibovici L. (2004). β-Lactam monotherapy versus β-lactam-aminoglycoside combination therapy for sepsis in immunocompetent patients: systematic review and meta-analysis of randomised trials BMJ 328 668 10.1136/bmj.38028.520995.63 . 14996699 PMC381218

[jmm000137-Peleg1] Peleg A. Y. Seifert H. Paterson D. L. (2008). *Acinetobacter baumannii*: emergence of a successful pathogen Clin Microbiol Rev 21 538–582 10.1128/CMR.00058-07 . 18625687 PMC2493088

[jmm000137-Petersen1] Petersen P. J. Labthavikul P. Jones C. H. Bradford P. A. (2006). *In vitro* antibacterial activities of tigecycline in combination with other antimicrobial agents determined by chequerboard and time-kill kinetic analysis J Antimicrob Chemother 57 573–576 10.1093/jac/dki477 . 16431863

[jmm000137-Petrosillo1] Petrosillo N. Ioannidou E. Falagas M. E. (2008). Colistin monotherapy vs. combination therapy: evidence from microbiological, animal and clinical studies Clin Microbiol Infect 14 816–827 10.1111/j.1469-0691.2008.02061.x . 18844682

[jmm000137-Pourcel1] Pourcel C. Minandri F. Hauck Y. D'Arezzo S. Imperi F. Vergnaud G. Visca P. (2011). Identification of variable-number tandem-repeat (VNTR) sequences in *Acinetobacter baumannii* and interlaboratory validation of an optimized multiple-locus VNTR analysis typing scheme J Clin Microbiol 49 539–548 10.1128/JCM.02003-10 . 21147956 PMC3043498

[jmm000137-Rodriguez1] Rodriguez C. H. Bombicino K. Granados G. Nastro M. Vay C. Famiglietti A. (2009). Selection of colistin-resistant *Acinetobacter baumannii* isolates in postneurosurgical meningitis in an intensive care unit with high presence of heteroresistance to colistin Diagn Microbiol Infect Dis 65 188–191 10.1016/j.diagmicrobio.2009.05.019 . 19748431

[jmm000137-Schultsz1] Schultsz C. Bootsma M. C. Loan H. T. Nga T. T. Thao T. P. Thuy T. T. Campbell J. Vien M. Hoa N. T. other authors (2013). Effects of infection control measures on acquisition of five antimicrobial drug-resistant microorganisms in a tetanus intensive care unit in Vietnam Intensive Care Med 39 661–671 10.1007/s00134-012-2771-1 . 23306583 PMC3607730

[jmm000137-Sheng1] Sheng W. H. Wang J. T. Li S. Y. Lin Y. C. Cheng A. Chen Y. C. Chang S. C. (2011). Comparative *in vitro* antimicrobial susceptibilities and synergistic activities of antimicrobial combinations against carbapenem-resistant *Acinetobacter* species: *Acinetobacter baumannii* versus *Acinetobacter* genospecies 3 and 13TU Diagn Microbiol Infect Dis 70 380–386 10.1016/j.diagmicrobio.2011.03.003 . 21558048

[jmm000137-Song1] Song J. Y. Cheong H. J. Choi W. S. Heo J. Y. Noh J. Y. Kim W. J. (2011). Clinical and microbiological characterization of carbapenem-resistant *Acinetobacter baumannii* bloodstream infections J Med Microbiol 60 605–611 10.1099/jmm.0.029439-0 . 21233298

[jmm000137-Soon1] Soon R. L. Nation R. L. Cockram S. Moffatt J. H. Harper M. Adler B. Boyce J. D. Larson I. Li J. (2011). Different surface charge of colistin-susceptible and -resistant *Acinetobacter baumannii* cells measured with zeta potential as a function of growth phase and colistin treatment J Antimicrob Chemother 66 126–133 10.1093/jac/dkq422 . 21081544 PMC3001852

[jmm000137-Sopirala1] Sopirala M. M. Mangino J. E. Gebreyes W. A. Biller B. Bannerman T. Balada-Llasat J. M. Pancholi P. (2010). Synergy testing by Etest, microdilution checkerboard, and time-kill methods for pan-drug-resistant *Acinetobacter baumannii* Antimicrob Agents Chemother 54 4678–4683 10.1128/AAC.00497-10 . 20713678 PMC2976112

[jmm000137-Tada1] Tada T. Miyoshi-Akiyama T. Kato Y. Ohmagari N. Takeshita N. Hung N. V. Phuong D. M. Thu T. A. Binh N. G. other authors (2013). Emergence of 16S rRNA methylase-producing *Acinetobacter baumannii* and *Pseudomonas aeruginosa* isolates in hospitals in Vietnam BMC Infect Dis 13 251 10.1186/1471-2334-13-251 . 23721359 PMC3680199

[jmm000137-Tan1] Tan T. Y. Lim T. P. Lee W. H. Sasikala S. Hsu L. Y. Kwa A. L. (2011). *In vitro* antibiotic synergy in extensively drug-resistant *Acinetobacter baumannii*: the effect of testing by time-kill, checkerboard, and Etest methods Antimicrob Agents Chemother 55 436–438 10.1128/AAC.00850-10 . 20956606 PMC3019682

[jmm000137-Taylor1] Taylor W. R. Nguyen K. Nguyen D. Nguyen H. Horby P. Nguyen H. L. Lien T. Tran G. Tran N. other authors (2012). The spectrum of central nervous system infections in an adult referral hospital in Hanoi, Vietnam PLoS One 7 e42099 10.1371/journal.pone.0042099 . 22952590 PMC3431395

[jmm000137-Wareham1] Wareham D. W. Gordon N. C. Hornsey M. (2011). *In vitro* activity of teicoplanin combined with colistin versus multidrug-resistant strains of *Acinetobacter baumannii* J Antimicrob Chemother 66 1047–1051 10.1093/jac/dkr069 . 21393131

[jmm000137-Wiegand1] Wiegand I. Hilpert K. Hancock R. E. (2008). Agar and broth dilution methods to determine the minimal inhibitory concentration (MIC) of antimicrobial substances Nat Protoc 3 163–175 10.1038/nprot.2007.521 . 18274517

[jmm000137-Woodford1] Woodford N. Ellington M. J. Coelho J. M. Turton J. F. Ward M. E. Brown S. Amyes S. G. Livermore D. M. (2006). Multiplex PCR for genes encoding prevalent OXA carbapenemases in *Acinetobacter* spp Int J Antimicrob Agents 27 351–353 10.1016/j.ijantimicag.2006.01.004 . 16564159

[jmm000137-Zhanel1] Zhanel G. G. Simor A. E. Vercaigne L. Mandell L. Canadian Carbapenem Discussion Group (1998). Imipenem and meropenem: comparison of *in vitro* activity, pharmacokinetics, clinical trials and adverse effects Can J Infect Dis 9 215–228 . 22346545 10.1155/1998/831425 PMC3250889

[jmm000137-Zusman1] Zusman O. Avni T. Leibovici L. Adler A. Friberg L. Stergiopoulou T. Carmeli Y. Paul M. (2013). Systematic review and meta-analysis of *in vitro* synergy of polymyxins and carbapenems Antimicrob Agents Chemother 57 5104–5111 10.1128/AAC.01230-13 . 23917322 PMC3811454

